# Keratin 23, a novel DPC4/Smad4 target gene which binds 14-3-3ε

**DOI:** 10.1186/1471-2407-11-137

**Published:** 2011-04-14

**Authors:** Sven-T Liffers, Abdelouahid Maghnouj, Johanna B Munding, René Jackstadt, Ulrike Herbrand, Thomas Schulenborg, Katrin Marcus, Susanne Klein-Scory, Wolff Schmiegel, Irmgard Schwarte-Waldhoff, Helmut E Meyer, Kai Stühler, Stephan A Hahn

**Affiliations:** 1 Medizinisches Proteom-Center, Ruhr-University Bochum - Zentrum fuer Klinische Forschung, Universitaetsstr. 150, 44780 Bochum, Germany; 2 Department of Internal Medicine, Molecular GI-Oncology, MGO, Ruhr-University Bochum - Zentrum fuer Klinische Forschung, Universitaetsstr. 150, 44780 Bochum, Germany; 3 Department of Pathology, Kliniken Bergmannsheil, University of Bochum, Buerkle-de-la-Camp-Platz 1, 44789 Bochum, Germany; 4 Department of Internal Medicine, Knappschaftskrankenhaus, IMBL, Ruhr-University Bochum, In der Schornau 23 - 25, 44892 Bochum, Germany; 5 Department of Gastroenterology and Hepatology, Kliniken Bergmannsheil, University of Bochum, Buerkle-de-la-Camp-Platz 1, 44789 Bochum, Germany

## Abstract

**Background:**

Inactivating mutations of SMAD4 are frequent in metastatic colorectal carcinomas. In previous analyses, we were able to show that restoration of Smad4 expression in Smad4-deficient SW480 human colon carcinoma cells was adequate to suppress tumorigenicity and invasive potential, whereas in vitro cell growth was not affected. Using this cellular model system, we searched for new Smad4 targets comparing nuclear subproteomes derived from Smad4 re-expressing and Smad4 negative SW480 cells.

**Methods:**

High resolution two-dimensional (2D) gel electrophoresis was applied to identify novel Smad4 targets in the nuclear subproteome of Smad4 re-expressing SW480 cells. The identified candidate protein Keratin 23 was further characterized by tandem affinity purification. Immunoprecipitation, subfractionation and immunolocalization studies in combination with RNAi were used to validate the Keratin 23-14-3-3ε interaction.

**Results:**

We identified keratins 8 and 18, heat shock proteins 60 and 70, plectin 1, as well as 14-3-3ε and γ as novel proteins present in the KRT23-interacting complex. Co-immunoprecipitation and subfractionation analyses as well as immunolocalization studies in our Smad4-SW480 model cells provided further evidence that KRT23 associates with 14-3-3ε and that Smad4 dependent KRT23 up-regulation induces a shift of the 14-3-3ε protein from a nuclear to a cytoplasmic localization.

**Conclusion:**

Based on our findings we propose a new regulatory circuitry involving Smad4 dependent up-regulation of KRT23 (directly or indirectly) which in turn modulates the interaction between KRT23 and 14-3-3ε leading to a cytoplasmic sequestration of 14-3-3ε. This cytoplasmic KRT23-14-3-3 interaction may alter the functional status of the well described 14-3-3 scaffold protein, known to regulate key cellular processes, such as signal transduction, cell cycle control, and apoptosis and may thus be a previously unappreciated facet of the Smad4 tumor suppressive circuitry.

## Background

The inactivation of the tumor suppressor DPC4 (deleted in pancreatic carcinoma, locus 4), also called SMAD4 or MADH4 is most frequently found in ductal pancreatic adenocarcinomas (approx. 50%) and metastatic colon carcinomas (approx. 30%) [1,2].

Smad4 and its homologs mediate signals from cytokines of the transforming growth factor-β (TGF-β) family from cell surface receptors to the nucleus where they regulate a diverse array of target genes involved in numerous biological functions including embryonic development, cell growth and differentiation, modulation of immune responses, and bone formation.

Ligand induced TGF-β receptor stimulation leads to the formation of a hetero-tetrameric receptor complex of two identical heterodimers, which is comprised of a type I and a type II receptor family member, each. Upon receptor activation the receptor-regulated Smads (R-Smads) can transiently interact with the type I receptor. R-Smads are thereby C-terminally phosphorylated by the receptor kinase and, once phosphorylated, they form a hetero-oligomeric complex with the "common-mediator" Smad4. This complex translocates into the nucleus, where it regulates the transcription levels of target genes by interacting with other transcription factors and by recruiting transcriptional co-activators or co-repressors [3]. In addition to R-Smads there are also inhibitory Smads (I-Smads) and other signaling molecules that feed into the TGF-β-Smad signalling cascade such as ERK, JNK and PKC [4].

This rather complex mode of target gene regulation involving Smad4 explains why currently more than 1000 genes were described to be either directly or indirectly regulated by Smad4 [5]. Furthermore, it is obvious that the cellular context will play a crucial role in defining the subset of Smad4 target genes relevant in a particular cellular differentiation state.

In the current work, we focused on colon carcinoma (SW480) cells to define potential Smad4 target genes involved in the neoplastic transformation process of this particular cell type. For the detailed investigation of Smad4's tumor suppressor functions, we stably re-expressed Smad4 via gene transfer in human Smad4-deficient SW480 tumor cells [6]. We were able to show that re-expression of Smad4 in these colon carcinoma cells was not sufficient to restore TGF-β responsiveness. These cells have accumulated a number of other oncogenic alterations in addition to and presumably prior to Smad4 inactivation [6,7], likely explaining the TGF-β resistance of Smad4 re-expressing derivatives. However, the re-expression of Smad4 in SW480 cells was sufficient to suppress tumor growth in vivo [6] confirming that these cells provide an adequate model to investigate Smad4 tumor suppressor function.

Here we focused on the study of the nuclear subproteome of Smad4 re-expressing SW480 cells in comparison to its Smad4 negative cells by establishing a subfractionation strategy coupled with the difference gel electrophoresis (DIGE) system and subsequent MALDI-MS-based peptide mass fingerprinting (PMF) to identify differentially expressed proteins.

Of the proteins which were identified as highly up-regulated in Smad4 re-expressing SW480 cells, we chose to follow-up on the KRT23 protein. Keratins are major structural proteins in epithelial cells. The keratin multigene family contains 50 individual members, which can be divided in two groups: (i) acidic forms and (ii) basic forms. The KRT9-23 belongs to the acidic group, whereas KRT1-8 are basic keratins [8]. Generally, one basic and one acidic keratin heterodimerize in order to form a functionally active intermediate filament. It has been postulated that the mechanical properties of these dimers are regulated by their specific keratin composition [9]. An association of differential expression patterns of keratins with tumor progression and the utility of measuring the keratin expression status for a differentiation between normal und tumor cells has been established [10-13]. Furthermore, it has been shown that in normal cellular senescence primary KRT8/18, i.e. keratins that are expressed first during tissue development, become partly substituted by secondary or later keratins (e.g. KRT20, KRT7) in a tissue dependent manner. This effect, however, is disrupted in transformed cells and thus expression profiling of keratins can be used for cancer diagnosis, i.e. the type of keratin detected allows the distinction between normal and cancerous tissue and for the definition of the type of carcinoma, even when the tumor is present as metastasis of unknown origin [14-16]. More recently, the classical structural role of keratins was extended to their involvement in cell signalling, stress response and apoptosis, mostly through their interaction with other proteins and/or their phosphorylation, glycosylation, transglutamination, caspase cleavage and ubiquitination state affecting keratin organization, distribution, turnover and function [17]. In colorectal microsatellite instable tumors the over-expression of KRT23 led to cell death. Due to this cellular response KRT23 was associated with a potential role as a tumor suppressor in this subset of colorectal cancers [18].

In this work we present data showing the interaction between 14-3-3 proteins and KRT23. The 14-3-3 protein family consists of 7 isoforms in mammalian cells (β, γ, ε, η, σ, τ, ξ) which form homo- and hetrerodimers with each other [19]. These dimers bind preferentially to the phosphorylated motifs RSXpSXP and RXXXpSXP present on most known 14-3-3 binding proteins [20]. The universal nature of this protein family has been shown using proteomic approaches [21-24]. The identified broad spectrum of 14-3-3 interacting partners illustrates the vast array of processes in which this protein family is involved. Based on the discovery of cytoplasmic sequestration of BAD a general sequestration model was postulated [25]. The core of this sequestration model is a nuclear export signal (NES)-like domain within the 14-3-3 molecules that bind to target proteins. Upon binding, NES sequences of the target proteins become exposed, which initiates translocation of the target protein from the nucleus into the cytoplasm thereby inhibiting the activity of the target molecule [26,27]. As numerous 14-3-3 interaction partners have been implicated in apoptosis and cell cycle regulation, it is not surprising that 14-3-3 proteins play a crucial role in carcinogenesis. Examples are the sequestration of CDC25s and p21 through 14-3-3ε [28-31].

Here, we report that re-expression of Smad4 in the SW480 cells strongly induces KRT23 expression, both at the transcript and protein level. A tandem affinity purification (TAP) assay with KRT23 as bait was performed to identify KRT23 interacting proteins. This assay revealed that 14-3-3ε is part of the KRT23-binding complex and that Smad4 re-expression and KRT23 up-regulation correlates with cytoplasmic translocalization of 14-3-3ε. In summary, our data suggest that Smad4-dependent KRT23 expression is probably important for the cellular localization of 14-3-3ε. This, in turn, will likely influence cellular signaling through 14-3-3 binding partners involved - amongst others - in cell cycle, intracellular trafficking/targeting and signal transduction; all these processes are known to be altered in carcinogenesis.

## Methods

Reagents and Cell culture - Monoclonal anti-tubulin (TUB2.1) and anti-FLAG (M2) antibodies were purchased from Sigma. Anti-lamin B (C-20) and anti-VSV-G (P5D4) antibodies were purchased from Santa Cruz Biotechnology, Inc. (Santa Cruz, CA). Human embryonic kidney (HEK) 293T cells as well as Smad4 negative and re-expressing derivatives of the colon carcinoma cell line SW480 [32] were cultured in DMEM media supplemented with 10% FBS and antibiotics.

DNA Constructs and Transient Transfection - cDNAs encoding the wild type FLAG-tagged KRT23 was amplified by PCR and ligated into the SalI-EcoRI site of the pMT2SM expression vector. The expression vector for VSV-G-tagged 14-3-3ε (pcDNA3-VSV-G-14-3-3ε) was kindly donated by H. Hermeking (LMU München, Germany). Cells were seeded onto 10 cm dishes 18 to 24 h prior to transfection and then transiently transfected at 60 to 70% confluence using FuGENE-6 transfection reagent (Roche Diagnostics). 48 h after transfection, cells were harvested for further assays.

Immunoprecipitation, Cell Fractionation and Immunoblotting - Smad4 re-expressing and Smad4 negative SW480 cells were lysed in ice cold RIPA buffer to prepare whole cell lysates. Lysates were cleared by centrifugation. HEK 293T cells were transfected with Flag-KRT23 and/or VSV-G-14-3-3ε expression vectors. Transfected cells were lysed in RIPA buffer to prepare whole cell lysates. Immunoprecipitation was performed using ANTI-FLAG M2-affinity agarose, with constant agitation overnight at 4°C. After extensive washes, proteins bound to the beads were eluted with denaturing Laemmli buffer. To isolate nuclear and cytoplasmic fractions, cells were washed twice with cold PBS and resuspended in 500 μl of hypotonic buffer (20 mM Tris-HCl pH 7.4, 5 mM MgCl_2_, 1.5 mM KCl, 0.1% NP-40, 50 mM NaF, 2 mM sodium orthovanadate, and protease inhibitors (Complete, Roche)). Cells were allowed to swell on ice for 10 min and then passed several times through a 26 1/2 gauge syringe needle, followed by centrifugation at 800 × g. The supernatants were further centrifuged at 15,000 × g to remove insoluble pellets, and the resulting supernatants were collected as the cytoplasmic fractions. The pellets were resuspended in 100 μl of TKM buffer (20 mM Tris-acetate pH 7.4, 50 mM KCl, 5 mM MgCl_2_, containing protease and phosphatase inhibitors). After centrifugation at 15,000 × g for 10 min, the supernatants were collected like the nuclear fractions. Whole cell lysates from SW480 cells, immunoprecipitated proteins and proteins derived from cell fractionation were subjected to SDS-PAGE and transferred onto polyvinylidinedifluoride membranes (Millipore), respectively. The membranes were incubated with the indicated antibodies followed by the corresponding secondary antibodies. The membranes were then developed with the ECL Western Blotting Detection System (Pierce).

Protein Isolation from Nuclear Fractions for 2D-PAGE Analyses - For nuclear fractionation and protein isolation 10 cell culture dishes (10 cm) each from Smad4 re-expressing and Smad4 negative SW480 cells were harvested. Cell breakage was done following swelling cells in hypotonic buffer (10 mM Tris, pH 7.4, 10 mM NaCl, 5 mM MgCl_2_, 0.5% Triton X-100) with a dounce homogenisator. Broken cells were centrifugated (1,000 × g, 4°C, 10 min) to collect a crude nucleus fraction. The nuclei containing pellet was transferred onto an iodixanol density step gradient (25%, 30%, 35% (v/v)). Separation over the gradient was done by centrifugation (16,000 × g, 4°C, 30 min) and the nucleus fraction was collected at the interphase located approximately between the 30% (v/v) and 35% (v/v) iodixanol. The collected fraction was subsequently diluted 1:2 in TKM buffer (20 mM Tris, pH 7.4, 250 mM Sucrose, 50 mM KCl, 5 mM MgCl_2_; supplemented with protease inhibitors). Resuspended nuclei were centrifugated (1,000 × g, 4°C, 20 min) and washed two additional times in TKM buffer. The harvested nuclei were snap frozen in liquid nitrogen and stored at -80°C until 2D-PAGE separation.

2D Gel Electrophoresis - Collected nuclear fractions were dissolved in DIGE buffer (30 mM Tris, pH 8.5, 2 M thiourea, 7 M urea, 4% CHAPS). For a minimal labeling of proteins with CyDyes (GE Healthcare) 50 μg protein was incubated with 400 pmol CyDye dissolved in anhydrous DMF p.a. After 30 min of incubation in the dark on ice 10 pmol lysine was added to stop the labeling reaction. Cy3 and Cy5 labels were used for Smad4 negative and Smad4 positive samples, respectively. As internal standard pooled lysates of nuclear fractions containing equal amounts of protein, each, from Smad4 re-expressing and negative cells were Cy2 labeled. After combining all three samples the mixture was applied on the IEF tube gel (20 cm × 0.7 mm). The IEF was performed with carrier ampholyte tube gels with an ampholyte mixture ranging from pH 2-11 for 8.05 kVh. After IEF tube gels were ejected, incubated in equilibration buffer (125 mM Tris pH 6.8, 40% (w/v) Glycerol, 3% (w/v) SDS, 65 mM DTT) for 10 min and subsequently applied on the second dimension gel (20 cm × 30 cm × 0.7 mm). The second dimension (SDS-PAGE) consisted of 15.2%T, 1.3%C polyacrylamide gels which were run in a Tris-Glycine buffer system. For protein identification the preparative gel format was: 20 cm × 1.5 mm for tube gels and 20 cm × 30 cm × 1.5 mm for SDS-PAGE.

Image Analysis - CyDye labeled proteins were visualized by Typhoon 9400 laser scanner (GE Healthcare) according to the user manual. Image analysis was performed with the DIA software tool (GE Healthcare) for single gel comparison and the Biological Variance Analysis (BVA) software tool (GE Healthcare) in case of matching gel sets. Parameters for significant changes in the spot pattern were set as follows: changes in the spot volume had to be two-fold, p-value of Student's T-test had to be ≤ 0.05 and the spot had to be detected in at least five of six gel sets.

Tryptic *in-gel *Digestion and MALDI-MS Protein Identification - For protein identification, silver stained protein spots or bands of interest were cut from a preparative gel, *in-gel *digested with trypsin (Promega, Madison, WI) and extracted as described previously [33].

For MALDI-MS target preparation peptides were concentrated via ZipTips™, eluted on the MALDI-target with 1.2 μL matrix solution (α-cyano-4-hydroxy cinnamic acid in ACN and 0,1% (v/v) TFA (1:2)) and analyzed using the UltraflexTM (Bruker Daltoniks). PMF spectra were acquired in positive mode with 20 kV target voltage and pulsed ion extraction of 17.25 kV. The reflector and detector voltage was set to 21 kV and 1.7 kV, respectively. Peak detection was carried out using FlexAnalysis 1.2 (Bruker Daltonics) with a S/N threshold of 2.5. The monoisotopic peptide mass values were transferred to ProteinScape™ (Bruker Daltonics) for subsequent protein identification via automated database analysis against the human IPI (V3.27; 67528 entries) by Profound (2002.03.01). To confirm the results obtained a randomized database combined with a normal IPI human database was performed. Searches were carried out with a mass tolerance of 100 ppm. Furthermore, propionamide (C, +71 Da) was set as a fixed and oxidation (M, +16 Da) as a variable modification and for the cleavage enzyme (trypsin) one missed cleavage site was allowed. Internal re-calibration of the obtained data was performed using a calibrant list and contained mass values were subsequently excluded prior database search. A Z score of 1.65 was used as threshold for the protein identification.

Protein Identification of TAP Purified Proteins - The Protein identification of TAP purified proteins were done by liquid chromatography (LC)-tandem MS (MS/MS). LC-MS/MS measurements were done with tryptic peptide extracts in 5% (v/v) FA by using a LC Packings Ultimate capillary LC system coupled to 4000 Q Trap™ (Applied Biosystems). Ionspray voltage was set to 3.0 - 3.2 kV in positive mode. EMS-scan was performed over a mass range from 400 to 1400 m/z. For MS/MS-scans the three highest signals were isolated and fragmentation was done with collision energy between 15-60 V according to m and z. MGF data were extracted from the raw data using Analyst 1.4.1 (Applied Biosystems) and Mascot (2.2.0) was used for database searches against the human IPI (V3.27; 67528 entries). To confirm the results, a randomized database combined with a normal IPI human database was performed. The mass accuracy was set to 1.5 Da for precursor ions and 0.5 Da for fragment ions. Further modification of cysteine by propionamide (C, +71 Da) was set as a fixed modification and oxidation of methionine (M, +16 Da) was set as a variable modification. One missed cleavage site for the tryptic digestion was allowed. Proteins were identified with three different peptides with an ion score of 32 and higher.

RNA Isolation and Northern Blotting - Total RNA was prepared from Smad4 re-expressing and negative cells as described previously [6]. Briefly, 5 μg total RNA was applied on 1% formaldehyde-agarose gel. Subsequently the gel was blotted onto Hybond N membranes (Amersham) with SSC buffer (0.15 M Sodium citrate pH 7.0, 1.5 M NaCl) and filters were hybridized at 50°C overnight with radiolabeled probes using the following oligonucleotides 5'-cgcgtcgaccaccatggactacaaggacgacgatgacaagaactccggacacagcttcag-3' and 5'-acagaattcaaca-ggcggaaactttcattg-3.

Production of Transgenic TAP-KRT23 SW480 Cells - The coding region of KRT23 was PCR-amplified from an image clone using the oligonucleotides 5'-caattgaactc cggacacagcttcag-3' and 5'-gtcgactcatgcgtgcttttggattt-3'. The resulting PCR-products were inserted into the MfeI, SalI site of retroviral vector pBabe-puro. The TAP affinity tag was amplified from pRAV-FLAG (kindly provided by X. Liu, University of Colorado, Boulder, CO, USA) with the oligonucleotides 5'-cgggatccatggcgcaacacgatgaagc-3' and 5'-gccaattgcttgtcatcgtcgtccttg-3'. Resulting PCR-fragments were digested with BamHI and MfeI and subsequently ligated into the pBabepuro vector containing the KRT23 sequence. The correct insert sequences of the newly generated pBabeTAP-KRT23 vector were confirmed by cycle sequencing analysis. Generation of the TAP-KRT23 expression cassette containing retrovirus was done in HEK 293T cells. Transfection of HEK 293T cells was done with FuGene (Roche) according to the manufacturer's instructions. For retroviral virus particle generation the packaging pHIT60 and the envelope pHITG plasmids were cotransfected together with the pBabeTAP-KRT23 retroviral vector. Viral infection of SW480 cells was done with filtered (0.45 μm pore size) retroviral particles released from 293T cells. Transduced SW480 cells were selected with 2 μg/mL puromycin (Sigma).

Tandem Affinity Purification (TAP) - SW480 cell lines stably expressing the TAP-tagged KRT23 protein were cultured in 14.5 cm culture dishes. Confluent SW480 cells from 15 dishes were used in each purification experiment. Before lysis, cells were washed three times with cold PBS and then lysed directly on the dish (50 mM Tris, pH 7.4, 150 mM NaCl, 5 mM EDTA, 1 mM DTT, 2% (v/v) Trition X-100, 50 mM NaF, 2 mM NaVO_4 _and protease inhibitor cocktail (complete mini, Roche)). After preclearing the samples (2 × 10 min, 1,000 × g, 4°C) the supernatants were incubated with 100 μL IgG-Sepharose (GE Healthcare) for 1 h at 4°C, followed by a washing step with cleavage buffer (50 mM Tris, pH 7.4, 150 mM NaCl, 5 mM EDTA, 1 mM DTT, 2% (v/v) Trition X-100, 50 mM NaF, 2 mM NaVO_4_) and subsequent TEV cleavage in 200 μl cleavage buffer with 100 units of TEV protease (Invitrogen). The TEV eluate was transferred onto 50 μL ANTI-FLAG M2-Agarose (Sigma) and incubated for 1 h at 4°C. After extensively washing the agarose beads were treated with 50 μl elution buffer (62.5 mM Tris pH 6.8, 2% (w/v) SDS, 10% (w/v) Glycerin) for 3 min at room temperature.

Confocal Laser Scanning Microscope Analysis - SW480 cells were seeded on glass coverslips. After 48 h cells were fixed in 3.5% (v/v) paraformaldehyde and permeabilized with 0.2% (v/v) Triton X-100. Before primary antibodies were applied (α-Flag M2 (mouse, Sigma); α-14-3-3ε (rabbit, Santa Cruz); α-β-tubulin (mouse, Sigma)) cells were blocked with 1% BSA. Secondary antibodies were labeled as followed: α-mouse IgG-FITC (Dianova) and α-rabbit IgG-TRITC (Dianova). Images of immunostained cells were generated with a Leica TCS SP2 confocal laser scanning microscope and processed with the Leica Confocal Software Version 2.5, build 1227.

ShRNA Down-Regulation of Keratin 23 in SW480 cells - Keratin 23 expression cells was silenced in SW480 cells by shRNAknock-down. The following oligonucleotide pairs KRT23-1506s: 5'-CCGGGCACGAAATCTGCTTTGGAAAGCTCGAGGTTTCCAAAGCAGATTT CGTGCTTTTTG-3', KRT23-1506as: 5'-AATTCAAAAAGCACGAAATCTGCTTTGGAA ACCTCGAGCTTTCCAAAGCAGATTTCGTGC-3' and KRT23-1010s: 5'-CCGGGCTCAG ATTATTCTTCTCATTGCTCGAGGAATGAGAAGAATAATCTGAGCTTTTTG-3'; KRT23-1010as: 5'-AATTCAAAAAGCTCAGATTATTCTTCTCATTCCTCGAGCAATGA GAAGAATAATCTGAGC-3' were annealed and subcloned into the EcoRI/AgeI site of the pLKO.1 puro vector (kindly provided by Sheila Stewart). Lentiviruses were made by transfecting packaging cells (HEK 293T) with a 3-plasmid system. DNA for transfections was prepared by mixing 12 μg pCMVΔRR8.2, 1 μg pHIT G and 12 μg pLKO.1 plasmid DNA with 62 μl of 2 M CaCl_2 _in a final volume of 500 μl. Subsequently 500 μl of 2x HBS phosphate buffer was dropwise added to the mixture and incubated for 10 min at RT. The 1 ml transfection mixture was added to 50% confluent HEK 293T cell seeded the day before into a 10 cm well plate. Cells were incubated for 16 h (37°C and 10% CO_2_), before the media was changed to remove remaining transfection reagent. Lentiviral supernatants were collected at 36 h post-transfection and for each infection 3 ml supernatant containing 4 μg/ml polybrene was immediately used to infect target cells seeded the day before in 6 well plates to reach 70% confluency on the day of infection. Cells were incubated for 24 h, and the media was changed to remove virus particles. To control infection rate a parallel infection under the identical conditions targeting the same cell line was prepared using a lentiviral GFP expression control vector (pRRLU6-CPPT-pSK-GFP, kindly provided by Sheila Stewart). 6 days after infection 2 μg/ml puromycin was added to the cell culture media. The knock-down efficiency was monitored by qRT-PCR using the SYBR Green Master Mix reagent (Applied Biosystems) and a 7700 Sequence Detector System (Applied Biosystems). Relative Expression changes are calculated relative to B2M (B2Ms: TGCTGTCTCCATGTTTGATGT ATCT and B2Mas: TCTCTGCTCCCCACCTCTAAGT). The KRT23 expression was measured using the following oligonucleotide pairs: KRT23s: GAACTGGAGCGGCAGA ACA and KRT23as: TTGATTCTTCCCGTGTCCCTT.

## Results

### Differential protein profiling of nuclear proteins from Smad4 deficient and Smad4 re-expressing SW480 cells

**Figure 1:**
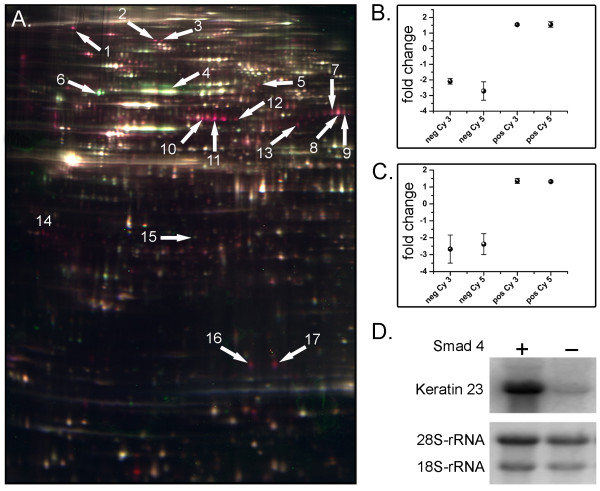
**Differential proteome analysis of Smad4 re-expressing and negative SW480 cells**. A) A representative example of the 2D-DIGE analyses is shown. Differentially expressed proteins are labeled by an arrow. The protein spot signals derived from the nuclear lysates of Smad4 negative cells are shown in green and those from Smad4 re-expressing cells in red. The protein names corresponding to each numbered spot are given in table 1. Spot numbers 10 and 11 mark the KRT23 spots. B) and C) Expression levels of the two KRT23 spots relative to the protein standard according to the dye swop experiment (neg., Smad4 negative SW480 cells; pos., Smad4 re-expressing SW480 cells) D) Expression of KRT23 analyzed by Northern blotting.

Smad4 re-expressing SW480 cells served as a model system to investigate the effects of the tumor suppressor Smad4 reconstitution on the nuclear protein composition of human colon carcinoma cells. Using 2D-DIGE analysis, we compared nuclear protein fractions derived from six independent replicates of Smad4 re-expressing and Smad4 negative SW480 cells, respectively (Figure 1A-C). We generated 3 sample sets each, derived from Cy3 labeled Smad4 re-expressing cell lysates and Cy5 labeled Smad4 negative cell lysates, whereas the second sample set (again three lysates, each for Smad4 re-expressing and negative cells) was labeled *vice versa *to minimize the identification of false positive protein spots. On average about 2000 spots were detected per gel. In the subsequent image analysis, using the DeCyder software (GE Healthcare), a total of 17 spots were identified that show a reproducible differential expression pattern. We considered intensity differences of factor two and above as significant. These 17 differentially expressed protein spots covered the entire pI and molecular weight range of the 2D-gels (Figure 1A). Of these 17 spots, 14 showed a higher and three a lower abundance in the nuclear fraction upon Smad4 re-expression. The subsequent protein identification by MALDI-MS revealed eight unique proteins (Table 1). The sequence coverage of these proteins ranged from 12.6 - 60.2% with Profound scores between 1.0 - 2.5 (1.3 being significant). The following proteins were found to be induced upon Smad4 re-expression: tumor rejection antigen (gp96), heterogeneous nuclear ribonucleoprotein R, eukaryotic translation elongation factor 1 alpha 1, KRT23, KRT18 and Cyclophilin A. In contrast, the amount of KRT8 and RbAp46 was found to be reduced in the nuclear fraction.

**Table 1:** Smad4 target proteins identified by DIGE coupled with MALDI-TOF-MS

Spot No	Accession No	Protein	Profound Score	Sequence coverage	No. of Peptides	
					matched	detected
1	gi|44890631	tumor rejection antigen (gp96) 1	2.4	14.6	15	21
3	gi|5031755	heterogeneous nuclear ribonucleoprotein R	2.5	23.4	12	20
4	gi|181573	cytokeratin 8	2.4	29.0	11	15
6	gi|4506439	retinoblastoma binding protein 7	2.4	26.4	12	20
7	gi|4503471	eukaryotic translation elongation factor 1 alpha 1	1.0	12.6	6	13
8	gi|4503471	eukaryotic translation elongation factor 1 alpha 1	1.8	20.3	8	20
9	gi|4503471	eukaryotic translation elongation factor 1 alpha 1	1.3	18.8	6	20
10	gi|27894339	cytokeratin 23	2.4	60.2	26	47
11	gi|27894339	cytokeratin 23	2.2	34.4	17	26
15	gi|30311	cytokeratin 18	2.4	22.9	12	21
16	gi|1633054	cyclophilin A	2.2	48.2	12	23
17	gi|1633054	cyclophilin A	1.6	51.2	7	20

Only spots with unequivocal identification by MALDI-TOF-MS are included in this table.

### Keratin 23 up-regulation occurs at the transcription level in a Smad4 dependent manner

As it is well known that the expression profiles of keratins change significantly during tumor progression and the consequences of these changes have dramatic effects on the morphology of the cells, we chose to study KRT23 further. About this particular keratin very little is known and our data clearly showed different expression levels in a Smad4-dependent context (Figure 1A, Spot 10 and 11), i.e. a threefold up-regulation in Smad4 re-expressing cells (Figure 1B, C). Subsequent Northern blot analysis of Smad4 re-expressing and negative SW480 cells confirmed the Smad4-dependent up-regulation already at the transcription level (Figure 1D). Having identified and confirmed the proteome data of the novel Smad4 target protein KRT23, we sought to identify protein interaction partners of this uncommon and poorly characterized keratin as an initial step to gather functional information for KRT23.

### Generation of stably expressing TAP-Keratin 23 cell lines

**Figure 2:**
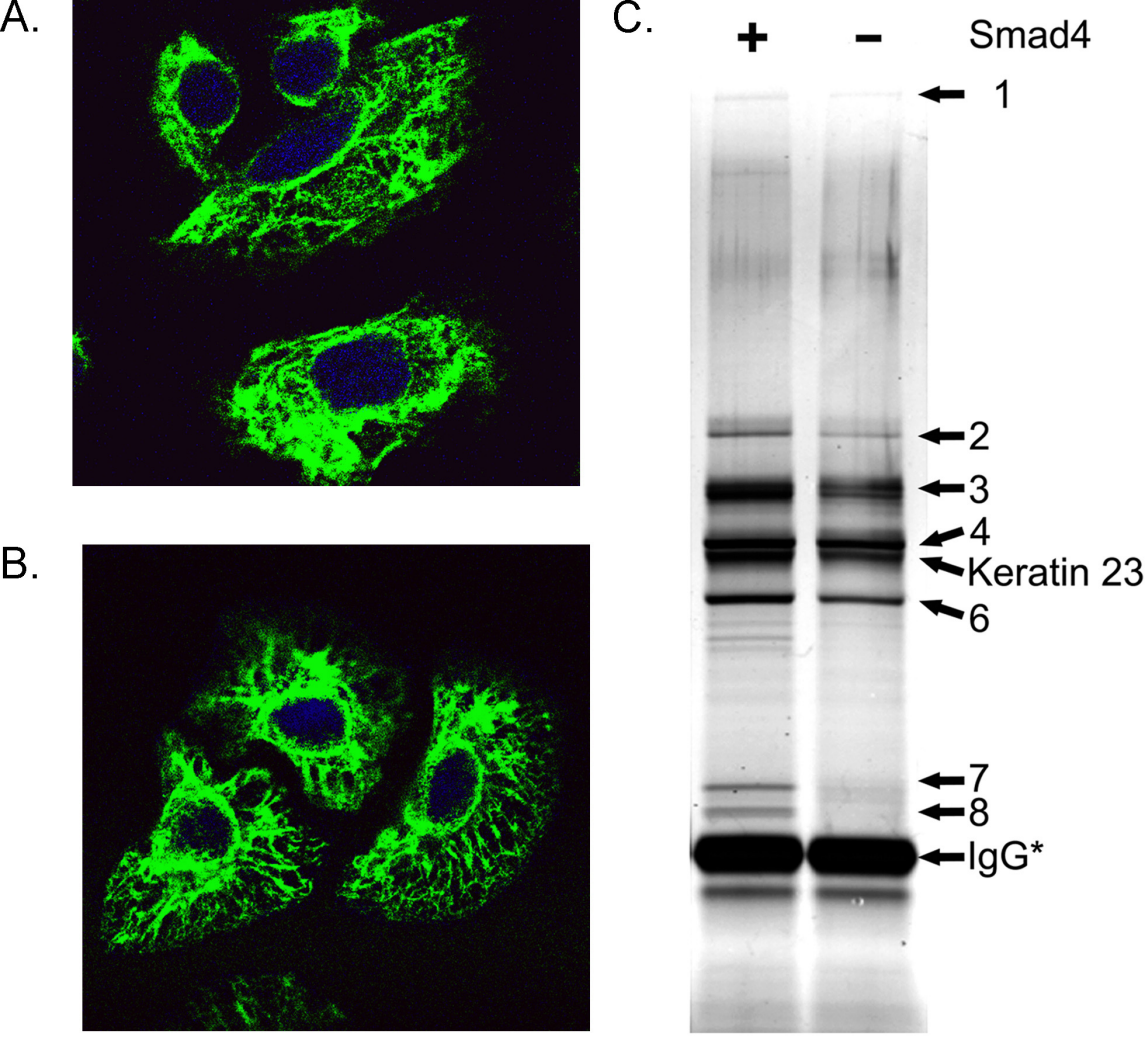
**Tandem affinity purification of Keratin 23**. Confocal immunofluorescence images of SW480 cells overexpressing KRT23. A) Smad4 re-expressing SW480 cells B) Smad4 negative SW480 cells. KRT23 was visualized by α-Flag M2 antibody. C) FLAG eluates from the tandem affinity purification experiments with TAP-KRT23 were separated on a standard 1D-PAGE and proteins visualized by silver staining. The protein names corresponding to each numbered band given in table 2.

To analyze the interaction partners of KRT23, we chose to perform a tandem affinity purification assay [34]. The N-terminal tag used for this experiment consists of a Flag-tag followed by two TEV cleavage sites and two Protein Z domains. Due to the generally poor transfection efficiency of SW480 cells with standard plasmid transfection strategies, we opted for retroviral gene transfer to generate Smad4 negative and Smad4 re-expressing SW480 cells which stably express the transgenic TAP-KRT23 protein. This approach offered a rapid and reliable means to purify native protein complexes and to identify the proteins by mass spectrometry [34]. The successful expression of the TAP-KRT23 protein was monitored by Western blotting (data not shown). In addition we analyzed the distribution of the over-expressed TAP-KRT23 by confocal microscopy. The immunofluorescence data revealed that the overexpressed protein is exclusively localized in the cytoplasm and surrounding the nucleus. Furthermore we observed a characteristic filamentous structure as expected for a member of the keratin family (Figure 2A, B).

### Tandem Affinity Purification of Keratin 23

KRT23 interaction partners were isolated following the TAP-strategy. Therefore, KRT23 protein complexes from Smad4 re-expressing and Smad4 negative cells were first affinity purified using IgG-Sepharose prior to TEV cleavage, followed by a second affinity purification step using immobilized ANTI-FLAG M2-Agarose. The FLAG eluates from the second purification step were separated by SDS-PAGE and the proteins visualized by silver staining. Five bands were present in both samples (Figure 2C, Bands 1 - 4 and 6), whereas two protein bands were unique to the Smad4 re-expressing cell line (Figure 2C, Bands 7 and 8). All bands were excised and *in-gel *digested with trypsin prior to analysis by nanoLC-MS/MS (Table 2). We were able to verify that the common bands contain the same set of proteins including, Plectin isoform 11, HSP70, HSP60, KRT8, KRT18. These proteins correspond to typical keratin-associated proteins. Two protein bands of approximately 30 kD, which were only detected in the Smad4 re-expressing cells, were identified as 14-3-3ε and 14-3-3γ, respectively.

**Table 2:** KRT23 interacting proteins identified by TAP followed by LC-MS/MS

Lane no.	NCBI accession no.	Protein identified	Peptides	Mascot Score
1	gi|40849944	plectin 1 isoform 11	ELYQQLQR	59
			LSVAAQEAAR	56
			FAEQTLR	46
			LAAIGEATR	46
			LTVDEAVR	44
			DLSELGSVR	41
			GLVEDTLR	41
			VSIYEAM*R	33
			DVAEVDTVR	32
2	gi|5729877	HSP70	NQVAM*NPTNTVFDAK	76
			VEIIANDQGNR	74
			DAGTIAGLNVLR	73
			LLQDFFNGK	45
			EIAEAYLGK	43
			NSLESYAFNM*K	38
			LIGDAAK	35
			ITITNDK	33
3	gi|31542947	HSP60	NAGVEGSLIVEK	85
			VTDALNATR	75
			VGGTSDVEVNEK	63
			VGLQVVAVK	62
			TVIIEQSWGSPK	59
			LSDGVAVLK	52
			DDAM*LLK	47
			EIGNIISDAM*K	45
			APGFGDNR	43
			IGIEIIK	41
			VGEVIVTK	41
			NAGVEGSLIVEK	33
4	gi|181573	cytokeratin 8	YEELQSLAGK	73
			LSELEAALQR	71
			SLDM*DSIIAEVK	65
			ASLEAAIADAEQR	59
			KLLEGEESR	57
			LESGM*QNM*SIHTK	56
			TEM*ENEFVLIK	55
			LQAEIEGLK	55
			ISSSSFSR	55
			SLDM*DSIIAEVK	52
			ELQSQISDTSVVLSM*DNSR	48
			TEISEM*NR	46
			LLEGEESR	45
			SRAEAESM*YQIK	44
			FASFIDK	44
			NKYEDEINKR	40
			DVDEAYM*NK	38
			SYTSGPGSR	36
			GELAIK	35
			AEAESM*YQIK	34
			YEDEINKR	33
			QLYEEEIR	33
			FLEQQNK	33
5	gi|20306864	cytokeratin 23	SALENM*LSETQSR	106
			EQSAAM*SQEAASPATVQSR	59
			DLEIEVEGLR	58
			LLEGESEGTR	57
			ATM*QNLNDR	56
			AITQETINGR	49
			MAVDDFNLK	47
			M*AVDDFNLK	47
			ISLSFTTR	44
			YENEHSFK	42
			LASYVEK	40
			APTVHGGAGGAR	38
			ALEEANM*K	35
6	gi|30311	cytokeratin 18	AQIFANTVDNAR	80
			LEAEIATYR	70
			TVQSLEIDLDSM*R	67
			ETM*QSLNDR	53
			LASYLDR	51
			LAADDFR	50
			KVIDDTNITR	46
			VIDDTNITR	45
			STFSTNYR	43
			SLETENR	42
			AQYDELAR	40
			YETELAM*R	38
			DWSHYFK	36
7	gi|67464424	14-3-3 ε	VAGM*DVELTVEER	95
			EAAENSLVAYK	84
			YLAEFATGNDR	72
			HLIPAANTGESK	54
			LAEQAER	50
			DSTLIM*QLLR	48
			YDEM*VESM*K	47
			IISSIEQK	44
			QM*VETELK	36
			NLLSVAYK	33
8	gi|21464101	14-3-3 γ	NVTELNEPLSNEER	71
			YLAEVATGEK	66
			LAEQAER	62
			DSTLIM*QLLR	58
			YDDM*AAAM*K	56
			NLLSVAYK	55
			VISSIEQK	42
			ATVVESSEK	40
			AYSEAHEISK	35

### Validation of Keratin 23-14-3-3ε-Interaction

**Figure 3:**
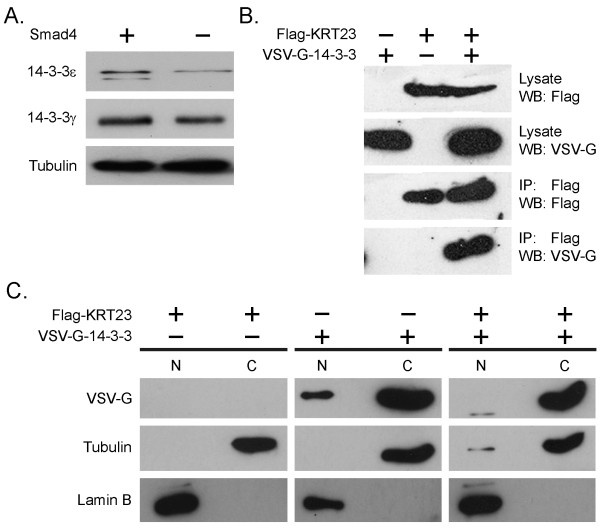
**Interaction of Keratin 23 with 14-3-3ε**. A) Endogenous 14-3-3ε and 14-3-3γ expression levels of Smad4 re-expressing and Smad4 negative SW480 cells. 20 μg whole cell lysates derived from Smad4 re-expressing and Smad4 negative cells were subjected to SDS-PAGE. Flag-tagged KRT23 and VSV-G-tagged 14-3-3ε were transfected into HEK 293T cells as indicated. B) Confirmation of KRT23-14-3-3ε interaction: cell lysates were immunoprecipitated with anti-Flag antibody and blotted as indicated. C) KRT23 expression leads to cytoplasmic sequestration of 14-3-3ε: following fractionation into cytoplasmic and nuclear fractions, proteins were subjected to Western blot analysis with the indicated antibodies. Anti-lamin B and anti-β-tubulin were used as marker proteins for the purity of the fractions. C, cytoplasmic fraction, N, nuclear fraction. A representative blot from three independent experiments is shown.

To further investigate the Smad4-dependent interaction between 14-3-3 and KRT23, we analyzed the endogenous expression of 14-3-3ε and 14-3-3γ in Smad4 re-expressing and Smad4 negative SW480 cells. The Western blot analysis showed that the expression of 14-3-3 proteins was reduced in Smad4 negative SW480 cells, compared to Smad4 re-expressing cells (Figure 3A). We chose to focus on the 14-3-3ε isoform for further analysis, because 14-3-3γ is known to form heterodimers with any other 14-3-3 family member whereas 14-3-3ε is only associated with the 14-3-3γ isoform. Using tagged proteins (Flag-KRT23 and VSV-G-14-3-3ε) in an immunoprecipitation experiment we were able to confirm our finding from the TAP-assay showing that KRT23 is an interaction partner of 14-3-3ε (Figure 3B). Having confirmed that 14-3-3ε is a KRT23 interaction partner, and taking into account previous findings by others showing that keratins are important for nuclear redistribution of 14-3-3 proteins [35,36], we went on to analyze whether KRT23 expression is able to modulate the cellular distribution of 14-3-3ε or not. HEK 293T cells overexpressing VSV-G-14-3-3ε were fractionated into cytoplasmic and nuclear fraction and the protein signal for VSV-G-14-3-3 could be recovered in both fractions. The co-expression of Flag-KRT23 and VSV-G-14-3-3 in turn lead to a decline of the VSV-G-14-3-3ε signal in the nuclear fraction, thus experimentally supporting a model where KRT23 expression is indeed influencing the cellular distribution of 14-3-3ε (Figure 3C).

### Cellular distribution of 14-3-3ε in SW480 cells depends on Keratin 23

**Figure 4:**
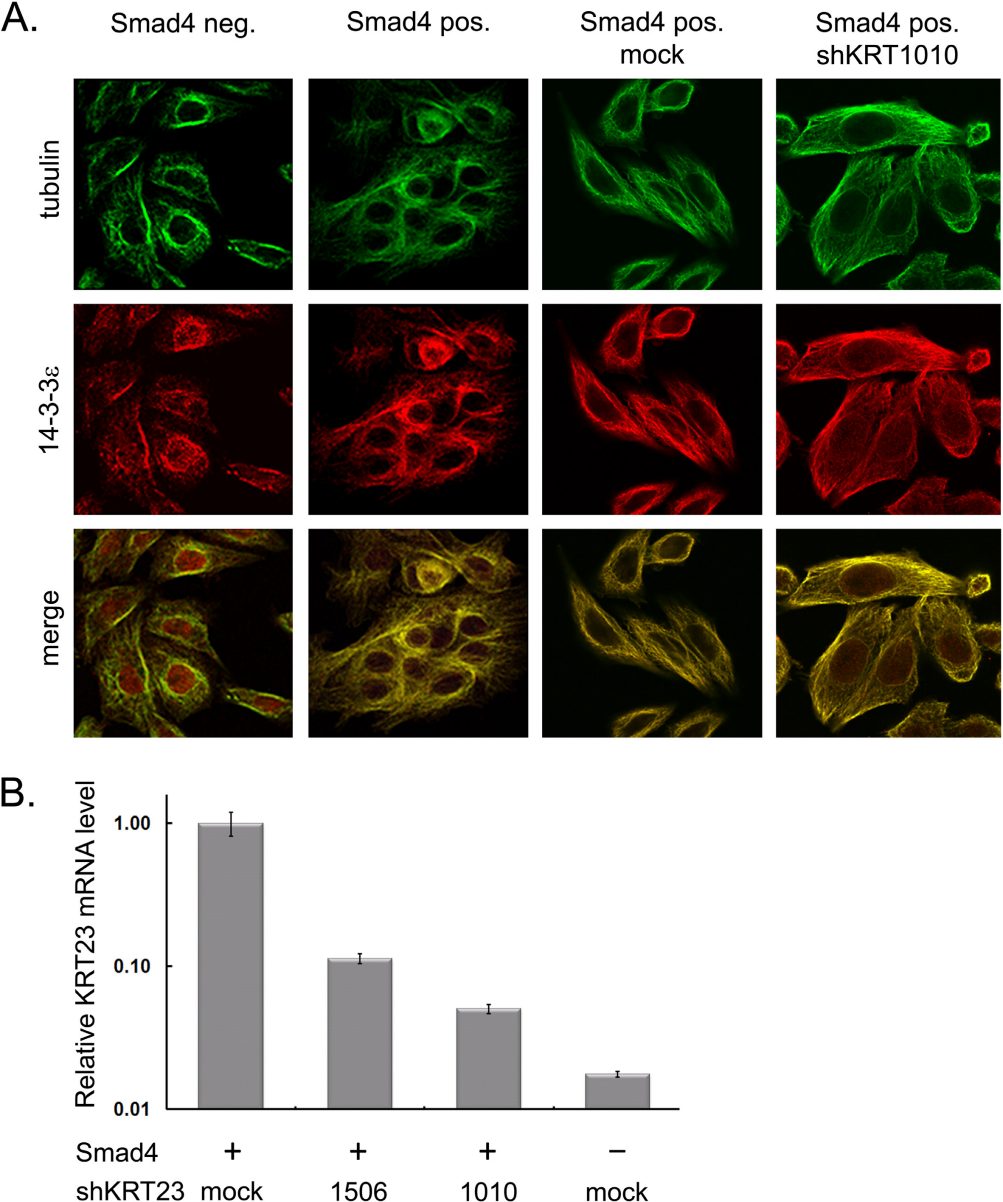
**Cellular localization of 14-3-3ε according to the Smad4 expression and KRT23 knock-down status of SW480 cells**. A) Confocal immunofluorescence images of SW480 cells. The cellular localization of 14-3-3ε was analyzed in Smad4 negative and Smad4 re-expressing SW480 cells. KRT23 knock-down was achieved by stable overexpression of shKRT1010 in Smad4 re-expressing cells. The cells were double stained with antibodies to 14-3-3ε (red) and the cytosolic marker tubulin (green). B) Validation of Keratin 23 knock-down efficiency. The Keratin 23 expression was monitored by quantitative RT-PCR in stably sh-KRT23 overexpressing SW480 cells as indicated. Each condition was measured in triplicates and normalized to B2M.

Having shown a link between KRT23 expression and 14-3-3ε localization, we further hypothesized that Smad4 re-expression in our SW480 cell model system (also shown to induce KRT23 expression) could have a similar effect on the endogenous cellular localization of 14-3-3ε. Indeed, confocal imaging revealed that in Smad4 re-expressing SW480 cells 14-3-3ε is more prominently localized in the cytoplasm, whereas in Smad4 negative cells (with a lower KRT23 expression level) 14-3-3ε showed a pronounced nuclear localization (Figure 4A). Next we wanted to test whether the Smad4 dependent nuclear exclusion of 14-3-3ε could be rescued by KRT23 knock-down experiments. The KRT23 expression was monitored by quantitative RT-PCR, because to the best of our knowledge the direct monitoring of the expression on the protein level is currently not possible due to the lack of a specific antibody. KRT23 expression was successfully reduced by both shRNA vector constructs; albeit to different levels depending on the KRT23 sequence targeted by the two different shRNA vector constructs (Figure 4B). In line with the reduced KRT23 expression following KRT23 knock-down, we observed a partial rescue of the nuclear 14-3-3ε localization which became visible in the shRNA vector construct shKRT1010, showing also the best KRT23 knock-down efficiency (Figure 4A).

## Discussion

### Identification of potential Smad4 targets

In this study we aimed to identify and characterize potential Smad4 target genes involved in colon carcinogenesis. In order to achieve this, we used our previously described model system of Smad4 deficient SW480 cells that re-express Smad4, thereby suppressing *in-vivo *tumor formation and invasive potential of SW480 cells [6]. Using high resolution 2D gel electrophoresis, we analyzed nuclear protein fractions from Smad4 re-expressing and negative SW480 cells. The following MALDI-MS analysis of differentially expressed protein spots revealed eight different proteins that can be grouped according to their function in i) gene regulatory proteins (hnRNPR; RpAp46; eEFa), ii) stress proteins (gp96; cyclophilin A) and iii) keratins (KRT8; KRT18 and KRT23). All of these proteins apart from gp96 and the keratins are considered nuclear proteins, whereas gp96 is an abundant protein located in the endoplasmic reticulum (ER). Gp96 is the ER-paralog of the cytosolic HSP90 with a role in housekeeping, i.e. maintenance of protein homeostasis in the secretory pathway [37]. The identification of an abundant ER protein is not surprising in this context because of the architecture of the nuclear envelope (NE): The NE consists of two membrane systems, the outer and inner nuclear membrane; the former being contiguous with the rough ER. Thus, we expected in our nuclear fractions protein contaminations derived from ER proteins as well as ribosomal proteins.

The observed presence of keratins in the nuclear fractions can be explained as a cytoplasmic contamination, by direct interactions between the nuclear lamina and cytosolic keratins [38] or by association of keratins with the outer nuclear membrane protein nesprin-3 via the cytoskeletal linker protein plectin [39]. The fact that our TAP-experiment also identified plectin, suggests the latter.

### Smad4 modulates Keratin 23 expression

Following the 2D-DIGE analyses which identified the altered expression of KRT23, we analyzed whether this increased expression is transcriptionally or post-transcriptionally regulated. Based on the Northern blot analysis showing a good correlation of KRT23 transcript and protein expression levels in SW480 cells (Figure 1D) it appears likely that KRT23 is mainly transcriptionally regulated. A subsequent test of eight human pancreatic carcinoma cell lines in which Smad4 expression was reconstituted by a retroviral expression vector revealed in three of the eight cell lines a similar Smad4-dependent KRT23 up-regulation (U. Herbrand, unpublished observation), supporting the notion that KRT23 expression levels can be modulated in two major gastrointestinal tumor types directly or indirectly through Smad4.

A connection between keratin expression and TGF-β signaling was previously demonstrated in dominant-negative TGF-β type II receptor mice having elevated K8/K18 protein levels [40]. In line with this mouse model data we found that reconstitution of Smad4, a key downstream component of the TGF-β signaling pathway, lead to the down-regulation of KRT8. However, in our model we found that Smad4 reconstitution increased in KRT18 expression.

In contrast to the increased KRT8/18 expression in mice in the absence of functioning TGFβ-signaling Zhang *et al*. showed in a human pancreatic carcinoma cell lines that sodium butyrate and trichostatin A treatment induces KRT23 expression at the mRNA level [41]. This effect could be inhibited by RNAi-mediated knock-down of p21 expression. Interestingly, Smad4 reconstitution in our SW480 model also led to a strong up-regulation of p21 (I. Schwarte-Waldhoff, unpublished observation). As p21 is a well described Smad4 target involved in the TGFβ-signaling pathway, these data would support a model, where Smad4 and p21 are upstream signaling components involved in the Smad4 dependent up-regulation of KRT23 described herein. Clearly, more work will be needed in order to elucidate the key proteins involved in our observed Smad4-dependend up-regulation of KRT23.

Bühler *et al*. recently reported that expression of KRT18 caused an induction of adhesion proteins and a regression of the malignant phenotype in KRT18 overexpressing breast carcinoma cells [42]. Smad4 loss is a late event during tumor progression and correlates with the development of a metastatic tumor in colon carcinogenesis [2,43], fitting well into a model where Smad4 induced expression of specific keratin types in colon cells may help to maintain the cell to cell junctions through desmosomes and hemidesmosomes and thus supporting an epithelial phenotype. Our data hint towards a model were Smad4 dependent KRT18 and KRT23 up-regulation and KRT8 down-regulation mediates a tumor suppressor effect presumably by playing a role in supporting the induction of the epithelial phenotype observed upon Smad4 reconstitution, which was also accompanied by an up-regulation of the invasion suppressor E-cadherin [32].

### Keratin 23-14-3-3ε interaction is Smad4 dependent

Interestingly, Hesse *et al*. noted in a phylogenetic tree analysis that KRT23 is an outstanding member of the type I keratins localized on chromosome 17 [44]. These data together with the increasing evidence that intermediate filaments are not only important as structural proteins but are also involved in modulating and controlling cellular signaling processes and apoptosis mostly through interaction with keratin associated proteins (KAPs) prompted us to initiate a more detailed study of KRT23 using the TAP methodology. Five of the seven identified KRT23 associated proteins (PLEC1, HSP70, HSP60, KRT8 and KRT18) were found both in Smad4 re-expressing and negative cells. Both, the 14-3-3ε and γ proteins were only identified in the TAP eluate of Smad4 re-expressing cells. However, we also found that 14-3-3ε and γ protein expression levels are reduced in Smad4 negative cells. Therefore, we hypothesis that this reduced protein level was sufficient to prevent the detection of 14-3-3 proteins by silver staining in our TAP assay. Nevertheless we would also like to point out that our data neither exclude nor clearly support the alternative possibility that Smad4 is directly modulating by any yet unknown mechanism KRT23-14-3-3 interaction. All of these proteins or their homologs have previously been shown to interact with other keratins [17], indicating that our experimental conditions were appropriate to identify KAPs. Furthermore, they provide evidence that although KRT23 is more distant to other KRT family members it is likely to share a number of interaction properties described for other keratins.

Liao *et al*. showed that KRT18 is able to bind 14-3-3η, ξ, and ε as well as HSP70. In their analyses KRT18-14-3-3 interaction was independent of HSP70 [45]. Ku *et al*. reported that keratin-14-3-3ζ interaction is able to modulate the cellular distribution of 14-3-3 proteins [36]. Similarly, it has been shown that keratin 17, is rapidly induced in wounded stratified epithelia and thus regulating cell growth through binding to the adaptor protein 14-3-3σ. Furthermore, phosphorylation of KRT17 was important for the redistribution of 14-3-3σ from the nucleus to the cytoplasm with concomitant stimulation of mTOR activity and cell growth [46]. These data prompted us to study the influence of Smad4 reconstitution and thus induction of KRT23 expression on the cellular distribution of 14-3-3ε in our model system.

### Keratin 23 modulates the distribution of 14-3-3

By Western blot analysis we showed that 14-3-3ε requires the presence of KRT23 for its cytoplasmic localization. In addition, laser confocal microscopy showed that the re-expression of the tumor suppressor Smad4 led both to an induction of KRT23 expression and cytoplasmic sequestration of 14-3-3ε. This cytoplasmic sequestration was partially released by KRT23 knock-down in Smad4 re-expressing cells. The close correlation between altering keratin expression and changing of 14-3-3 distribution was previously shown for hepatocytes [35]. In agreement with the hypothesis of Tzivion *et al*. that intermediate filament proteins alter signaling pathways through 14-3-3 sequestration, it seems plausible that Smad4-dependent up-regulation of KRT23 and/or KRT18 may have a similar effect on 14-3-3 localization [47].

Margolis *et al*. provided evidence for a pivotal role of keratins as potential drivers of mitotic entry of cells [48]. Due to the preferred binding of 14-3-3 to keratin in the cytoplasm (designated by Margolis *et al*. as "the 14-3-3 sink"), the cytoplasmic availability of keratins and its binding to 14-3-3 ensures that the 14-3-3 cargo proteins are released enabling them to control downstream cellular processes. Furthermore, it has been shown that 14-3-3 is able to modulate cytoplasmic localization of target proteins by directing them toward the CRM1-mediated nuclear export pathway [26,49,50]. These data together with our data are suggestive for a model where KRT23-14-3-3 interaction can mediate the relocalization of nuclear ligands by several mechanisms that ensure cytoplasmic sequestration of the bound 14-3-3 complex.

## Conclusions

In summary, we provide evidence that Smad4 is able to induce either directly or indirectly KRT23 expression. Furthermore, we were able to identify several novel KRT23 interacting proteins among them 14-3-3ε and γ. Finally, we found that KRT23 expression in Smad4 re-expressing cells is able to cytoplasmic sequestration of 14-3-3ε. These findings together with the known signal transduction modulator function of 14-3-3 family members suggests that our observed new regulatory circuit of Smad4 dependent KRT23 up-regulation which in turn modulates the cytoplasmic sequestration of 14-3-3 is a previously unknown facet of the tumor suppressive response we observed upon Smad4 re-expression in our colon cancer model. Therefore, it will be interesting to determine in future experiments, which are the potential proteins whose cellular localization and activity are modulated by the Smad4 dependent KRT23 sequestration of the 14-3-3 complex to the cytoplasm in our SW480 model system and what are their cellular functions in colon tumorigenesis.

## Competing interests

The authors declare that they have no competing interests.

## Authors' contributions

STL, RJ, UH and TS performed the experimental work. STL, AM, KS and SAH carried out data interpretation. STL, AM, KS and SAH participate in the experimental design. JBM, KM, SKS, WS, ISW and HEM revised the manuscript critically for important intellectual content. SAH participated in its design and coordination. All authors read and approved the final manuscript.

## Pre-publication history

The pre-publication history for this paper can be accessed here:

http://www.biomedcentral.com/1471-2407/11/137/prepub
